# Differentiating Between Obturator and Inferior Epigastric Arterial Injury in Traumatic Pelvic Hemorrhage: A Case Report

**DOI:** 10.7759/cureus.44593

**Published:** 2023-09-02

**Authors:** Joey Almaguer, Dylan Murray, Matthew Murray, Richard Murray

**Affiliations:** 1 Department of Radiology, Texas Tech University Health Sciences Center School of Medicine, Amarillo, USA; 2 Department of Surgery, University College Dublin, Dublin, IRL; 3 Department of Surgery, Royal College of Surgeons, Dublin, IRL; 4 Department of Diagnostic and Interventional Radiology, Northwest Texas Healthcare System, Amarillo, USA

**Keywords:** hematoma, pelvic trauma, embolization, hemorrhage, corona mortis, accessory obturator, aberrant obturator, inferior epigastric, pubic branches

## Abstract

The pubic branches of the inferior epigastric and obturator arteries are subject to injury from pelvic trauma or surgery within the retropubic space. Such injuries can result in severe internal hemorrhage that can lead to hemodynamic instability if not adequately controlled. Due to their anatomical proximity and anastomosis, it is critical to determine which artery is hemorrhaging in order to provide accurate embolization. In the presented case, a geriatric patient suffered a fall from standing height that resulted in bilateral and multiple pelvic fractures. CT angiography of the abdomen demonstrated active left-sided pelvic hemorrhage and a resultant 10 cm anterior extraperitoneal hematoma, likely exacerbated by existing anticoagulant usage. Urgent embolization of the inferior epigastric artery was performed in addition to multiple transfusions. The patient recovered without any procedural complications and was later discharged for rehabilitation.

## Introduction

The inferior epigastric artery (IEA) originates as the second branch of the external iliac artery and then bifurcates into a superior division and an inferior division. The superior division of the IEA travels into the rectus sheath at the level of the accurate line, whereas the inferior division of the IEA travels along the superior pubic ramus and terminates with pubic branches. These pubic branches then anastomose with the pubic branches of the obturator artery (OA).

The OA normally originates as the first branch from the anterior division of the internal iliac artery. Due to its high clinical relevance, it is important to distinguish between three additional anatomical variations of the OA: the aberrant (replaced) obturator artery (AbOA), accessory (anomalous) obturator artery (AcOA), and the arterial corona mortis (ACM) [1]. 

The AbOA has no connection to the internal iliac artery or its branches, instead arising solely from the IEA, external iliac artery, or common femoral artery. The AcOA is similar in origin to the AbOA, yet the key difference is that an OA arising from the internal iliac artery is also present. On the other hand, the ACM is an anastomotic connection between the OA and the IEA or external iliac artery. The difference between an AcOA and an ACM is that an ACM is an anastomosis, whereas an AcOA shares no connection with the existing OA [1]. Because the names and trajectories of these anatomical variations are very similar, they are sometimes used interchangeably, contributing to further confusion.

The AbOA travels over the superior ramus and pierces the obturator fascia and membrane to supply all of the structures attributed to the OA. Studies that report AbOA prevalence are limited, variable in their conclusions, and difficult to determine, especially with mixed usage of anatomical terminology. A couple of studies reported a prevalence of 11% and 19% [2,3]. However, a smaller study reported a prevalence as high as 39%, with six of the seven AbOAs originating from the IEA and one of the seven AbOAs originating from the external iliac artery [4]. More cadaveric or angiographic studies are needed to determine the true prevalence of the AbOA as a separate subtype of obturator anomaly.

The course of the AcOA follows a similar trajectory to the AbOA, with the only difference being the presence of an OA that stems from the anterior division of the internal iliac artery. To our knowledge, there are no published studies that describe the prevalence of AcOA in the general population, likely due to its minimal frequency of occurrence. The rarity of this subtype of obturator anatomical variation may be attributed to the lack of necessity for redundant blood supply to obturator territory. It is currently unclear in this scenario whether the pubic branches are more likely to originate from the OA or from the AcOA.

The ACM (“crown of death”) owes its ominous translation to its location adjacent to the superior pubic ramus, making it susceptible to injury by traumatic pelvic fracture or iatrogenic surgical laceration. This normal arterial variant traverses posteriorly over the superior pubic ramus and in between the lacunar ligament medially and the iliopubic eminence laterally. The estimated incidence of ACM varies depending on the report and the method of measurement used. In angiographic studies, the incidence is reported to be as low as 14% to as high as 33%. However, in cadaveric studies, the incidence is reported to be as low as 19% to as high as 65% [5]. One theory for this discrepancy is that the older population is more susceptible to atherosclerotic occlusion of the internal and external iliac arteries, making the development of collateral vascular connections more probable [6]. A meta-analysis described a 49.3% incidence of corona mortis, with 41% being venous and 17% being arterial [7]. The ACM was found to be more prevalent in females and more commonly unilateral than bilateral (2:1) [8].

Because the internal and external iliac systems are two high-volume blood reservoirs, injury to the ACM can result in extensive and prolonged hemorrhage. With the retropubic space commonly trafficked by numerous surgical specialists (e.g., gynecologists, urologists, orthopedic surgeons, and general surgeons), iatrogenic laceration is another form of possible injury [1]. Examples of implicated iatrogenic causes include traditional or laparoscopic hernia repair, acetabular surgeries, oncological pelvic dissections, angioplasty, and surgery for paravaginal defects [7,9]. Pelvic trauma, specifically fractures of the pubic body and superior pubic ramus, is another important cause of ACM injury [10]. The following case describes an injury to the pubic branches of the IEA secondary to the traumatic pubic body and superior pelvic ramus fracture, requiring catheter-guided embolization for control of internal hemorrhaging. 

## Case presentation

An 86-year-old female with a past medical history of atrial fibrillation on Eliquis and Plavix presented to the emergency department complaining of left lower abdominal pain after suffering a ground-level fall at her home. She described pain in her right shoulder and right hip but did not report loss of consciousness. Her blood pressure on arrival was 126/73 mmHg, with a hemoglobin of 10.6 g/dL and a hematocrit of 30%. Prothrombin time/international normalized ratio (PT/INR) was 13.7/1.22 and activated partial thromboplastin time (aPTT) was 24.2 s. After a few hours, her blood pressure decreased to 70/36 mm Hg, consistent with traumatic hemorrhagic shock. Her Eliquis and Plavix were held, and she subsequently received 1 L of crystalloid bolus and prothrombin complex concentrate, which increased her blood pressure to 138/63 mmHg. 

**Figure 1:**
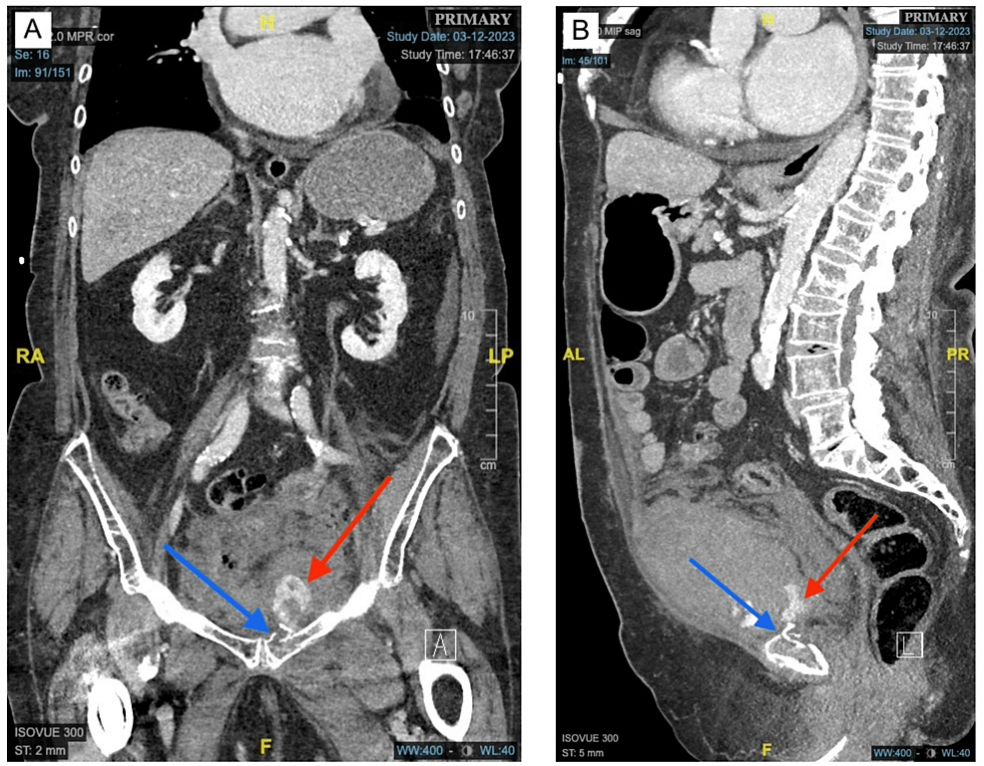
CT angiography showing active pelvic bleeding. (A) Coronal view of the left pubic body and superior pubic ramus fracture (blue arrow) and resultant active internal bleeding (red arrow). (B) Sagittal view of left pubic body fracture (blue arrow) with resultant active internal bleeding (red arrow). The nondisplaced fractures of the right inferior pubic ramus and right sacral wing are not shown in these images.

**Figure 2:**
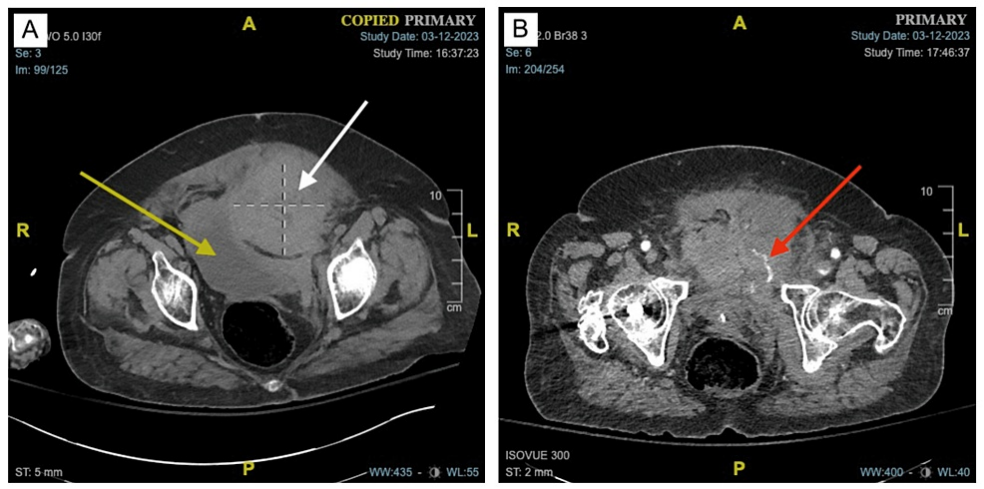
CT showing an anterior pelvic hematoma secondary to a traumatic pelvic fracture. (A) CT without contrast demonstrating a 10 x 9.8 x 9.6 cm extraperitoneal hematoma (white arrow) that is exerting a mass effect on the urinary bladder (yellow arrow). (B) CT angiography showing active internal bleeding into the hematoma (red arrow).

A pan-CT was ordered, which revealed a mildly comminuted, nondisplaced fracture of the left pubic body and superior pubic ramus as well as a nondisplaced fracture of the right inferior pubic ramus and right sacral wing (Figure 1). Secondary to the fracture, the CT also demonstrated a 10 x 9.8 x 9.6 cm extraperitoneal low anterior pelvic hematoma left of the midline. The hematoma was exerting an extrinsic mass effect, displacing the urinary bladder posteriorly, inferiorly, and to the right (Figure 2). CT angiography of the abdomen and pelvis revealed active bleeding into the large anterior pelvic hematoma immediately superior to the left pubic body where the comminuted fracture and a few displaced cortical fragments were located.

The patient underwent pelvic angiography via right percutaneous femoral artery puncture under local 1% lidocaine anesthesia and ultrasound guidance. A Bentson wire was passed into the aorta and a pigtail catheter was placed in the infrarenal abdominal aorta for flush angiography of the pelvis, which demonstrated active left pelvic bleeding. Selective angiography of the left internal iliac artery was performed during which contrast refluxed into the left external iliac artery and again active bleeding was identified superior to the left pubic ramus fracture. 

Initially, it was thought that the bleeding must be coming from an internal iliac branch, which is the case in the vast majority of pelvic trauma; however, super-selective angiography of several internal iliac branches in the vicinity, including the left internal pudendal and left inferior gluteal artery, did not demonstrate active bleeding. This inability to find the bleeding artery led to a closer inspection of the initial general pelvic angiogram and the initial left internal iliac angiogram, during which there had been reflux into the left external iliac artery.

It was now recognized that the hemorrhages were arising from the left external iliac system via the pubic branches of the IEA and that there was an absence of normal OA from the left internal iliac system. Selective catheterization and angiography of the left external iliac artery confirmed active bleeding arising from the pubic branches of the IEA. Because there was an absence of the usual OA originating from the internal iliac artery, it was initially thought that the bleeding was originating from an AbOA. 

**Figure 3:**
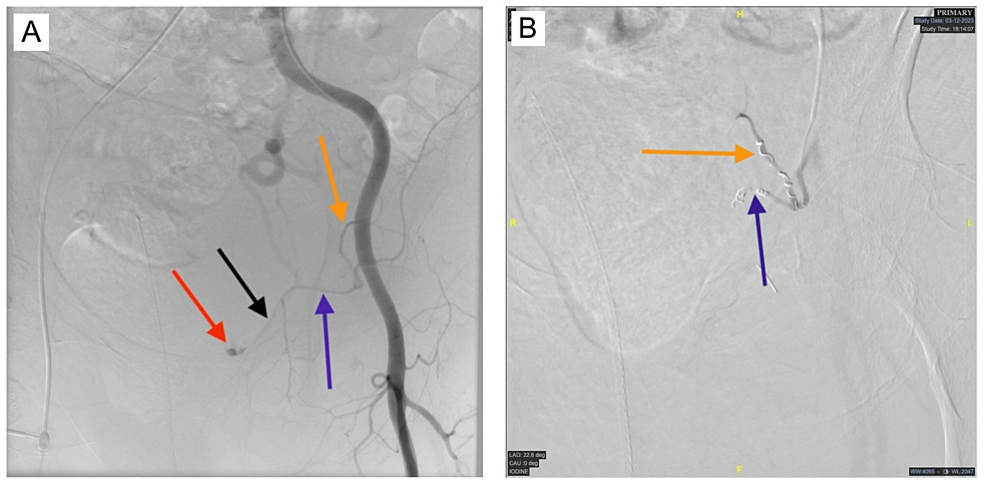
Fluoroscopic images of internal pelvic bleeding and embolization treatment. (A) The inferior division of the IEA (purple arrow) is seen originating from the IEA (orange arrow). The pubic branches of the IEA (black arrow) extend the length of the superior pubic ramus terminating at the pubic body. Extravasation of contrast indicating active bleeding (red arrow) can be visualized in close proximity to the location of the pelvic fractures. (B) Coil and Gelfoam embolization were performed in the IEA (orange arrow) and its inferior division (purple arrow), preventing further internal hemorrhage. IEA: inferior epigastric artery.

The left IEA and its inferior division were catheterized using a 2.8 Fr. Progreat microcatheter and 0.21 glide wire delivered coaxially through an over-the-horn 5 Fr. Judkins catheter, and embolization of the proximal IEA and its inferior division was performed with microcoils and Gelfoam. Post-embolization angiography of the left IEA stump demonstrated occlusion of the left IEA with no further active bleeding (Figure 3). 

During the procedure, the patient received one unit of packed red blood cells (PRBCs) and one unit of fresh frozen plasma, with no complications noted. Post-procedure complete blood count (CBC) examination in the surgical ICU yielded a hemoglobin level of 6.2 g/dL, and she received two additional units of PRBCs and two units of platelets. The patient stayed in the surgical ICU for three additional days for monitoring, and she did not require any further transfusions or surgical interventions. 

Venous thromboembolism prophylaxis consisted of a sequential compression device, and pain was well managed with analgesics. She was able to ambulate weight bearing as tolerated and was subsequently discharged home after three weeks of rehabilitation. 

## Discussion

Because of its adjacent proximity to the pubic body and superior pubic ramus, the pubic branches of the IEA are susceptible to injury in cases of pelvic trauma. Once the injury is identified, it is important to differentiate between a hemorrhage originating from the pubic branch of OA and a hemorrhage originating from the pubic branch of the IEA. There are only a handful of cases that report internal hemorrhage as a result of injury to the pubic branches of the IEA [10-15], yet treatment with transarterial embolization remains the most effective solution in both scenarios. It is also possible that there is injury to both the pubic branches of the OA and the pubic branches of the IEA. In this scenario, catheter-guided embolization of both the OA and IEA would be necessary. 

**Figure 4:**
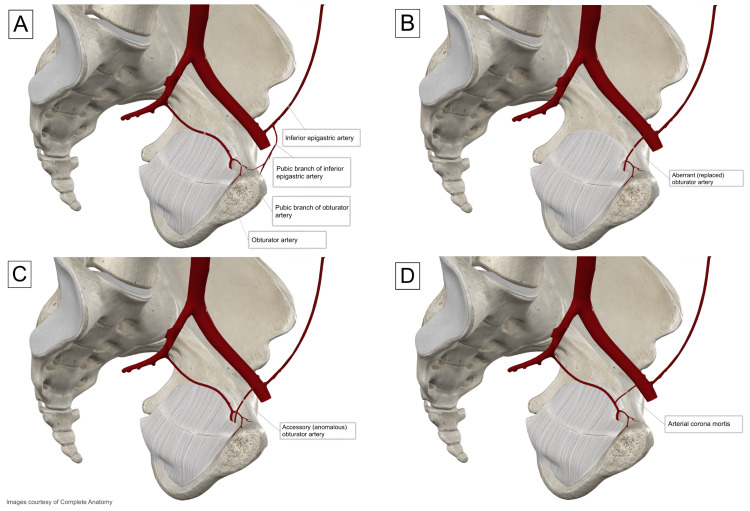
Illustration of the four general anatomical variations of the OA. (A) The normal distribution of the OA originates from the anterior division of the internal iliac artery, with the pubic branch of the OA anastomosing with the pubic branch of the IEA. (B) The AbOA is the only OA present and shares no connection to the internal iliac system. (C) The AcOA is similar to the AbOA, yet there is a normal OA present. (D) The ACM is similar to the AcOA; however, the ACM is an anastomotic connection between the internal and external iliac systems. OA: obturator artery; IEA: inferior epigastric artery; AbOA: aberrant (replaced) obturator artery; AcOA: accessory (anomalous) obturator artery; ACM: arterial corona mortis. Image credits: Made by Joey Almaguer with complete anatomy.

Similarly to the IEA, the pubic branches of the OA are susceptible to pelvic trauma and iatrogenic injury. If such an injury occurs and internal hemorrhaging results, knowledge of the anatomical variations of the OA is important for localization of the bleeding site and effective treatment (Figure 4). For example, if an ACM is present, embolization of the internal iliac system alone would not be sufficient to achieve hemostasis from a hemorrhage of the pubic branches of the OA. Similarly, the OA has been documented to originate on rare occasions from the posterior division of the internal iliac artery or from one of the branches of either division, which can further complicate attempts at embolization. 

In the described case, initial difficulty localizing the source of the pelvic bleeding led to a closer inspection of the angiograms revealing an active hemorrhage originating from the inferior division of the IEA. In addition, the absence of the typical OA originating from the internal iliac artery was also identified on angiography. It is unclear as to why an OA originating from the internal iliac artery was not observed. Possible reasons for this could be atherosclerotic blockage, procedural complications, an OA deriving from the posterior division of the internal iliac system, or others [2]. Because the internal pelvic hemorrhage identified on CT imaging was closer to the pubic body, the source of the bleeding likely originated from the pubic branches of the IEA rather than an AbOA. Evidence for this can be attributed to the lack of vascular distribution expected from an AbOA. 

## Conclusions

The vast anastomotic network within the pelvis can make localizing the direct source of internal pelvic hemorrhage difficult to determine. In cases of internal pelvic hemorrhage arising from pubic branches, it is important to recognize the IEA, OA, or anatomical variations as possible sources of bleeding. If originating from the pubic branches of the IEA, embolization of the IEA and its inferior division is effective at achieving hemostasis and preventing further hemorrhaging. 
